# Supplementary figures and images for: Development of polymorphic EST-SSR markers and characterization of the autotetraploid genome of sainfoin (Onobrychis viciifolia)
Source: PeerJ. 2019 Mar 26;7:e6542. doi: 10.7717/peerj.6542 (PMC6440460; doi:10.7717/peerj.6542)

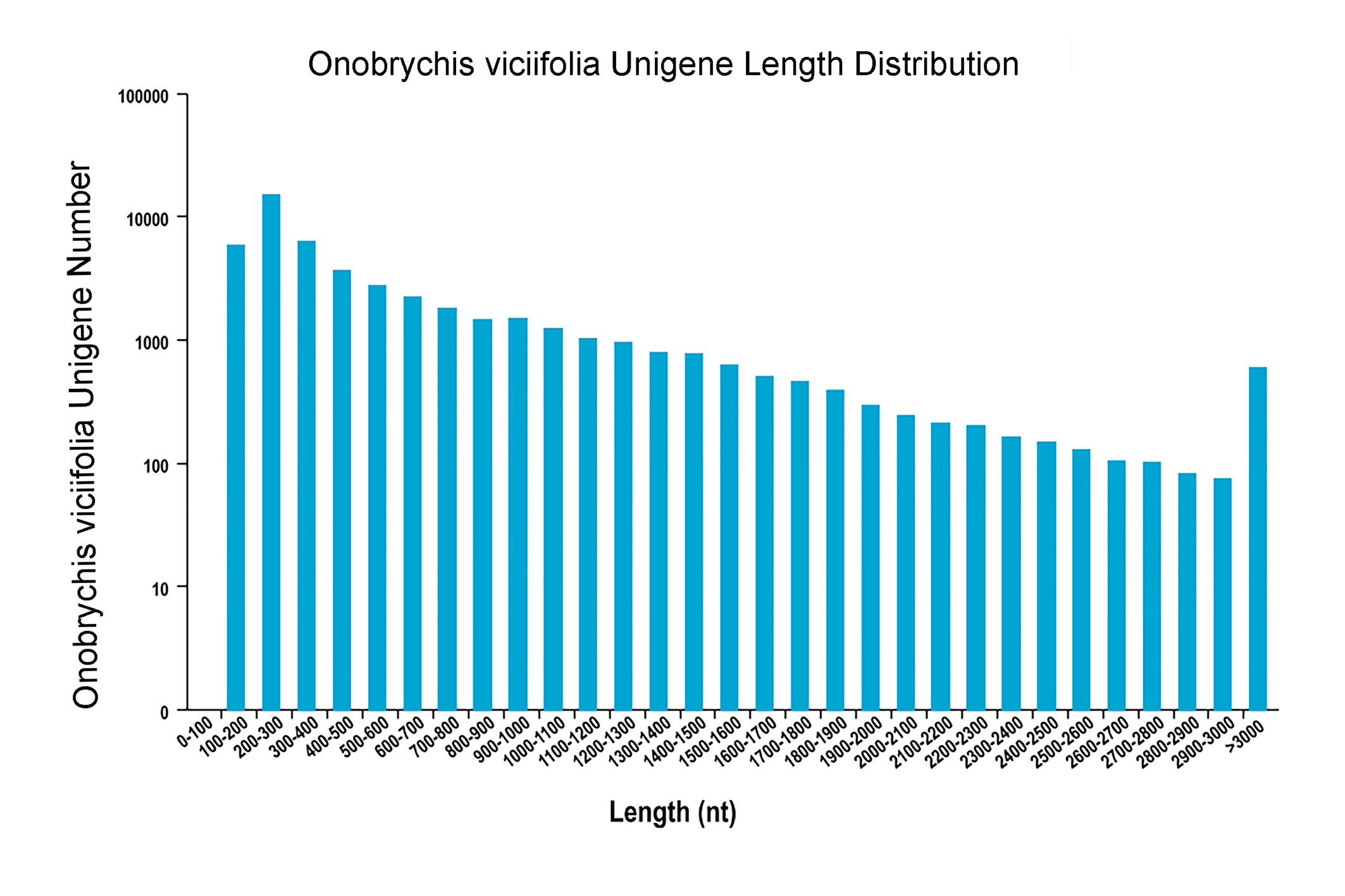

Supplement: Figure S1 — The x-axis represents the size of all unigenes, and the y-axis represents the number of unigenes within a certain range of length. [file peerj-07-6542-s001.png]

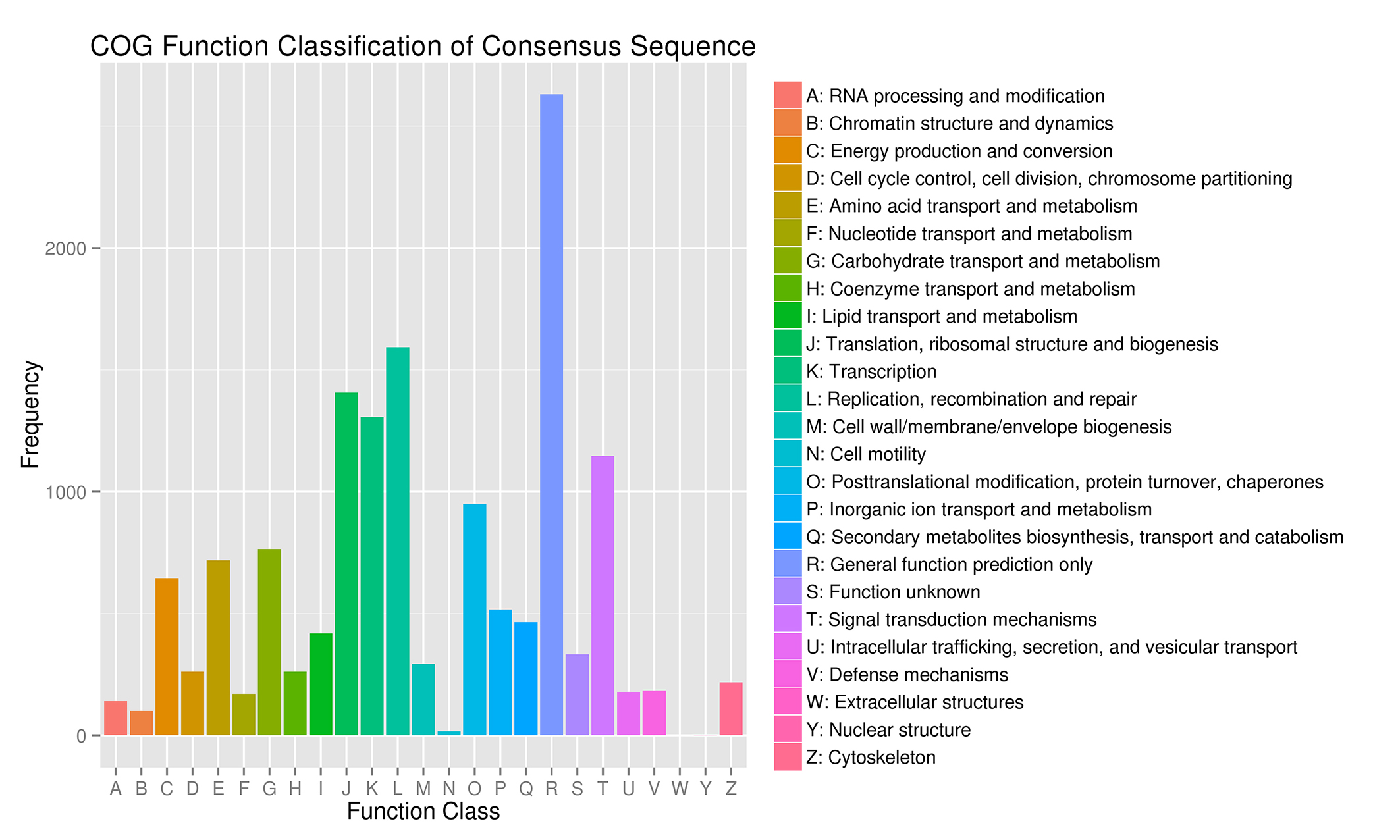

Supplement: Figure S2 — The y-axis indicates the number of unigenes in a specific functional cluster. A–Z and different colors indicate different COG categories. [file peerj-07-6542-s002.png]

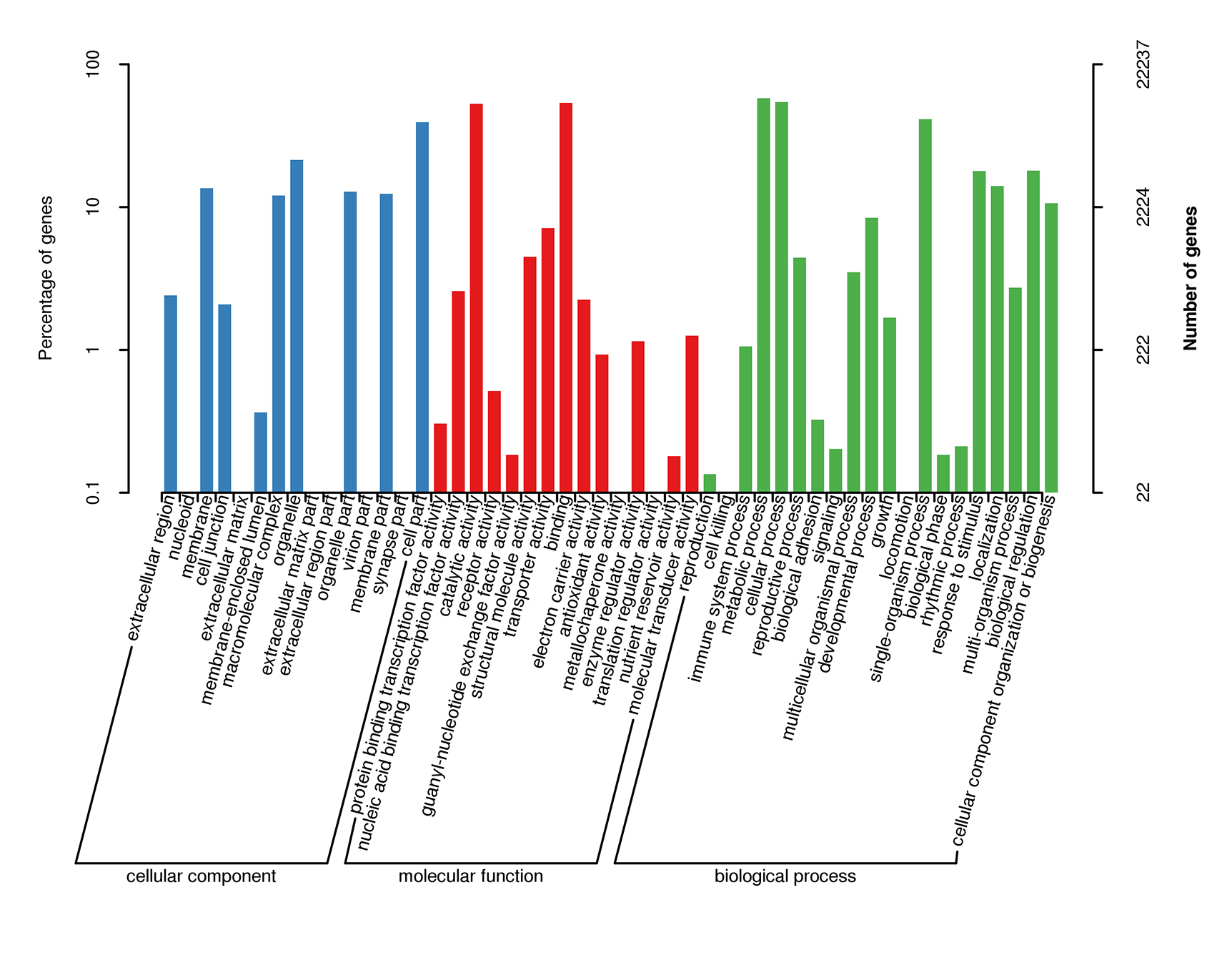

Supplement: Figure S3 — The y-axis on the right indicates the number of genes in a category. The y-axis on the left indicates the number of genes in a specific category. [file peerj-07-6542-s003.png]

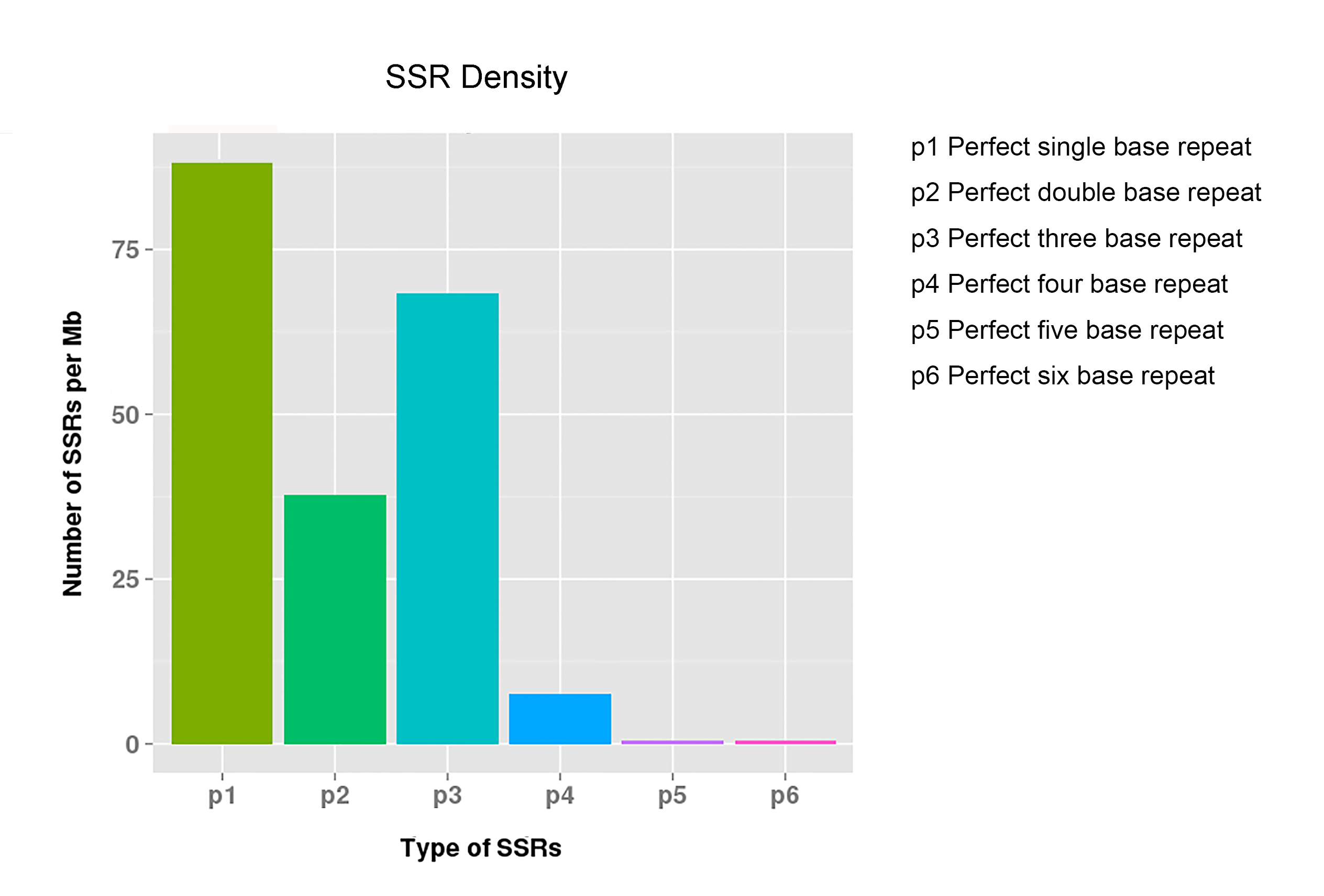

Supplement: Figure S4 [file peerj-07-6542-s004.png]

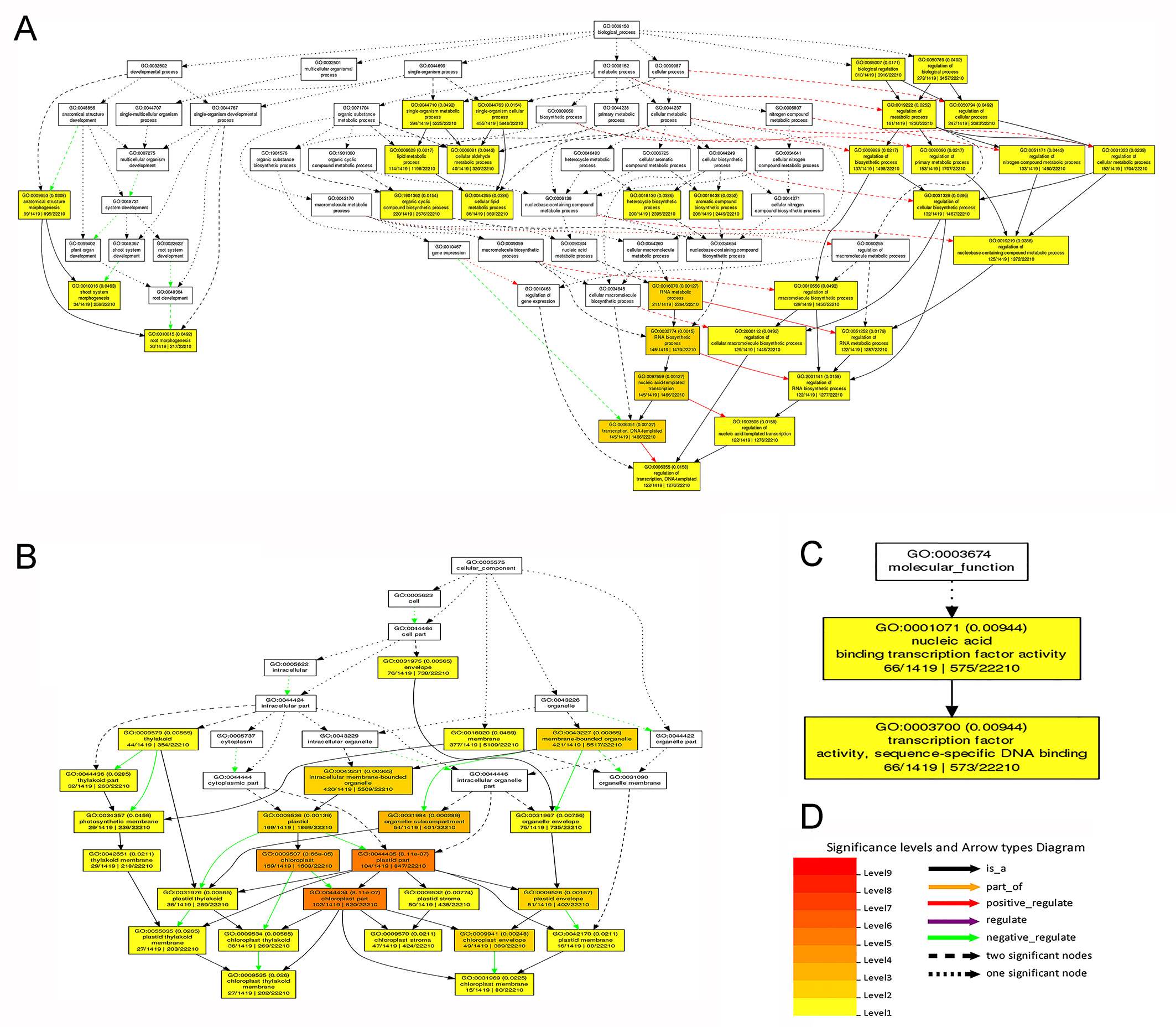

Supplement: Figure S5 — (A) Biological process. (B) Cellular component. (C) Molecular function. (D) legend. The color scale shows the p-value cut-off levels for each biological (Cellular) process. A deeper color denotes a more significant biological pathway. [file peerj-07-6542-s005.png]

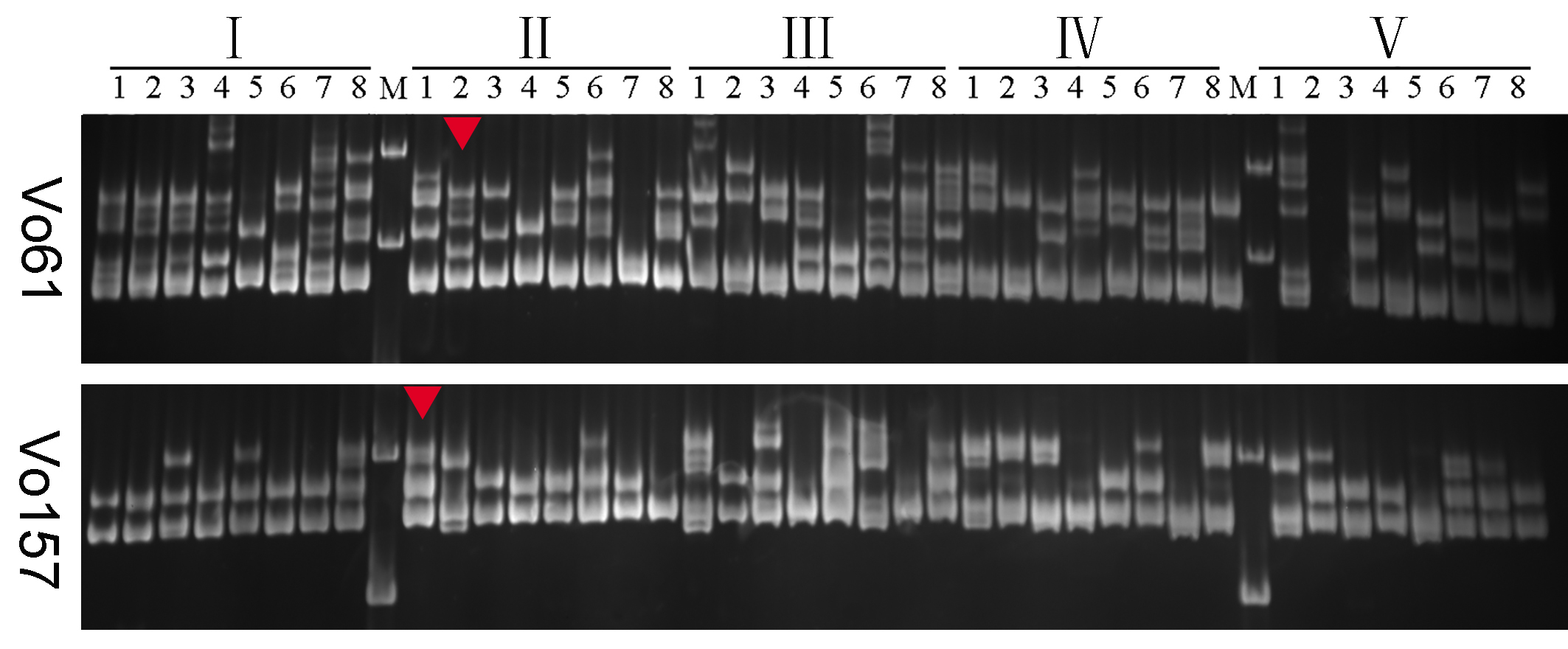

Supplement: Figure S6 — The letter M denotes a molecular marker of 300 and 200 bp size (top to bottom). The red triangle indicates the individual plant used to cut the polyacrylamide gel, Vo61selected No. 2 individual from site 2, Vo157 selected No.1 individual from site 2. [file peerj-07-6542-s006.png]
