# Supplementary material for: Development of polymorphic EST-SSR markers and characterization of the autotetraploid genome of sainfoin (Onobrychis viciifolia)
Source: PeerJ. 2019 Mar 26;7:e6542. doi: 10.7717/peerj.6542 (PMC6440460; doi:10.7717/peerj.6542)
Supplement: Table S1 [file peerj-07-6542-s007.docx]

**Supplemental Table S1 Detailed information of EST-SSRs based on the number of nucleotide repeat units.**

| **Repeats** | **5** | **6** | **7** | **8** | **9** | **10** | **11** | **12** | **13** | **14** | **≥15** | **Total** | **Percentage (%)** | |
| --- | --- | --- | --- | --- | --- | --- | --- | --- | --- | --- | --- | --- | --- | --- |
| A/T | - | - | - | - | - | 1,365 | 625 | 268 | 216 | 152 | 269 | 2,895 | 42.88% |  |
| C/G | - | - | - | - | - | 3 | 2 | 2 | 2 | 0 | 2 | 11 | 0.16% |  |
| AC/GT | - | 38 | 29 | 13 | 9 | 4 | 1 | - | - | - | - | 94 | 1.39% |  |
| AG/CT | - | 130 | 99 | 72 | 50 | 43 | 23 | 2 | - | - | - | 419 | 6.21% |  |
| AT/AT | - | 56 | 32 | 16 | 7 | 5 | 1 | - | - | - | - | 117 | 1.73% |  |
| CG/CG | - | - | 1 | - | - | - | - | - | - | - | - | 1 | 0.01% |  |
| CA/TG | - | 55 | 16 | 10 | 3 | 2 | 3 | - | - | - | - | 89 | 1.32% |  |
| GA/TC | - | 138 | 83 | 73 | 59 | 63 | 30 | 2 | - | - | - | 448 | 6.64% |  |
| GC/GC | - | 1 | - | - | - | - | - | - | - | - | - | 1 | 0.01% |  |
| TA/TA | - | 56 | 27 | 20 | 10 | 3 | 2 | - | - | - | - | 118 | 1.75% |  |
| AAC/GTT | 44 | 27 | 4 | 1 | - | - | - | - | - | - | - | 76 | 1.13% |  |
| AAG/CTT | 116 | 51 | 21 | - | - | - | - | - | - | - | - | 188 | 2.78% |  |
| AAT/ATT | 53 | 21 | 9 | 1 | - | - | - | - | - | - | - | 84 | 1.24% |  |
| ACC/GGT | 65 | 16 | 4 | - | - | - | - | - | - | - | - | 85 | 1.26% |  |
| ACG/CGT | 4 | - | 2 | 1 | - | - | - | - | - | - | - | 7 | 0.10% |  |
| ACT/AGT | 30 | 13 | 6 | 1 | - | - | - | - | - | - | - | 50 | 0.74% |  |
| AGC/CTG | 32 | 17 | 2 | - | - | - | - | - | - | - | - | 51 | 0.76% |  |
| AGG/CCT | 50 | 12 | 5 | - | - | - | - | - | - | - | - | 67 | 0.99% |  |
| ATG/CAT | 52 | 24 | 8 | 1 | - | - | - | - | - | - | - | 85 | 1.26% |  |
| ACA/TGT | 45 | 19 | 6 | 1 | - | - | - | - | - | - | - | 71 | 1.05% |  |
| AGA/TCT | 111 | 60 | 17 | - | - | - | - | - | - | - | - | 188 | 2.78% |  |
| ATA/TAT | 49 | 17 | 10 | - | - | - | - | - | - | - | - | 76 | 1.13% |  |
| CCG/CGG | 5 | 1 | - | - | - | - | - | - | - | - | - | 6 | 0.09% |  |
| CAA/TTG | 67 | 36 | 7 | 2 | - | - | - | - | - | - | - | 112 | 1.66% |  |
| CAC/GTG | 19 | 6 | 1 | - | - | - | - | - | - | - | - | 26 | 0.39% |  |
| CAG/CTG | 17 | 7 | 2 | - | - | - | - | - | - | - | - | 26 | 0.39% |  |
| CAT/ATG | 22 | 9 | 2 | - | - | - | - | - | - | - | - | 33 | 0.49% |  |
| CCA/TGG | 41 | 19 | 7 | - | - | - | - | - | - | - | - | 67 | 0.99% |  |
| CGA/TCG | 9 | 5 | - | 2 | - | - | - | - | - | - | - | 16 | 0.24% |  |
| CGC/GCG | 3 | 2 | - | - | - | - | - | - | - | - | - | 5 | 0.07% |  |
| CTA/TAG | 23 | 8 | 3 | - | - | - | - | - | - | - | - | 34 | 0.50% |  |
| CTC/GAG | 60 | 21 | 8 | - | - | - | - | - | - | - | - | 89 | 1.32% |  |
| GAA/TTC | 151 | 74 | 26 | - | - | - | - | - | - | - | - | 251 | 3.72% |  |
| GAC/GTC | 8 | 5 | - | 1 | - | - | - | - | - | - | - | 14 | 0.21% |  |
| GAT/ATC | 29 | 19 | 2 | - | - | - | - | - | - | - | - | 50 | 0.74% |  |
| GCA/TGC | 49 | 18 | 6 | - | - | - | - | - | - | - | - | 73 | 1.08% |  |
| GCC/GGC | 6 | - | - | 2 | - | - | - | - | - | - | - | 8 | 0.12% |  |
| GCT/AGC | 19 | 12 | 1 | - | - | - | - | - | - | - | - | 32 | 0.47% |  |
| GGA/TCC | 67 | 26 | 9 | - | - | - | - | - | - | - | - | 102 | 1.51% |  |
| GTA/TAC | 57 | 26 | 4 | - | - | - | - | - | - | - | - | 87 | 1.29% |  |
| CTC/GAG | 10 | 12 | - | - | - | - | - | - | - | - | - | 22 | 0.33% |  |
| TAA/TTA | 41 | 20 | 7 | 1 | - | - | - | - | - | - | - | 69 | 1.02% |  |
| TCA/TGA | 76 | 23 | 12 | 1 | - | - | - | - | - | - | - | 112 | 1.66% |  |
| AAAC/GTTT | 3 | - | - | - | - | - | - | - | - | - | - | 3 | 0.04% |  |
| AAAG/CTTT | 14 | - | - | - | - | - | - | - | - | - | - | 14 | 0.21% |  |
| AAAT/ATTT | 7 | - | - | - | - | - | - | - | - | - | - | 7 | 0.10% |  |
| AACA/TGTT | 2 | - | - | - | - | - | - | - | - | - | - | 2 | 0.03% |  |
| AACC/GGTT | 3 | 1 | - | - | - | - | - | - | - | - | - | 4 | 0.06% |  |
| AAGA/TCTT | 25 | 2 | - | - | - | - | - | - | - | - | - | 27 | 0.40% |  |
| AAGC/GCTT | 1 | - | - | - | - | - | - | - | - | - | - | 1 | 0.01% |  |
| AATA/TATT | 4 | 1 | - | - | - | - | - | - | - | - | - | 5 | 0.07% |  |
| AATC/GATT | 4 | - | - | - | - | - | - | - | - | - | - | 4 | 0.06% |  |
| AATT/AATT | 1 | - | - | - | - | - | - | - | - | - | - | 1 | 0.01% |  |
| ACAA/TTGT | 3 | - | - | - | - | - | - | - | - | - | - | 3 | 0.04% |  |
| ACAG/CTGT | 2 | - | - | - | - | - | - | - | - | - | - | 2 | 0.03% |  |
| ACAT/ATGT | 3 | 1 | - | - | - | - | - | - | - | - | - | 4 | 0.06% |  |
| ACGA/TCGT | 1 | - | - | - | - | - | - | - | - | - | - | 1 | 0.01% |  |
| ACTA/TAGT | 1 | - | - | - | - | - | - | - | - | - | - | 1 | 0.01% |  |
| ACTC/GAGT | 8 | - | - | - | - | - | - | - | - | - | - | 8 | 0.12% |  |
| AGAA/TTCT | 9 | 1 | - | - | - | - | - | - | - | - | - | 10 | 0.15% |  |
| AGAC/GTCT | 3 | 1 | - | - | - | - | - | - | - | - | - | 4 | 0.06% |  |
| AGAT/ATCT | 5 | 1 | - | - | - | - | - | - | - | - | - | 6 | 0.09% |  |
| AGCA/TGCT | 1 | - | - | - | - | - | - | - | - | - | - | 1 | 0.01% |  |
| AGCC/GGCT | 1 | - | - | - | - | - | - | - | - | - | - | 1 | 0.01% |  |
| AGGA/TCCT | 4 | 1 | - | - | - | - | - | - | - | - | - | 5 | 0.07% |  |
| AGTT/AACT | 1 | - | - | - | - | - | - | - | - | - | - | 1 | 0.01% |  |
| ATAA/TTAT | 1 | - | - | - | - | - | - | - | - | - | - | 1 | 0.01% |  |
| ATAC/GTAT | 2 | - | - | - | - | - | - | - | - | - | - | 2 | 0.03% |  |
| ATAG/CTAT | 2 | 1 | - | - | - | - | - | - | - | - | - | 3 | 0.04% |  |
| ATCA/TGAT | 4 | 1 | - | - | - | - | - | - | - | - | - | 5 | 0.07% |  |
| ATCC/GGAT | 3 | - | - | - | - | - | - | - | - | - | - | 3 | 0.04% |  |
| ATTA/TAAT | 4 | - | - | - | - | - | - | - | - | - | - | 4 | 0.06% |  |
| ATTC/GAAT | 1 | - | - | - | - | - | - | - | - | - | - | 1 | 0.01% |  |
| ATTG/CAAT | 1 | - | - | - | - | - | - | - | - | - | - | 1 | 0.01% |  |
| CAAA/TTTG | 5 | - | - | - | - | - | - | - | - | - | - | 5 | 0.07% |  |
| CACT/AGTG | 2 | 1 | - | - | - | - | - | - | - | - | - | 3 | 0.04% |  |
| CATT/AATG | 1 | - | - | - | - | - | - | - | - | - | - | 1 | 0.01% |  |
| CCAA/TTGG | 1 | - | - | - | - | - | - | - | - | - | - | 1 | 0.01% |  |
| CCAC/GTGG | 1 | - | - | - | - | - | - | - | - | - | - | 1 | 0.01% |  |
| CCAT/ATGG | 1 | - | - | - | - | - | - | - | - | - | - | 1 | 0.01% |  |
| CCTT/AAGG | 3 | - | - | - | - | - | - | - | - | - | - | 3 | 0.04% |  |
| CTAA/TTAG | 1 | - | - | - | - | - | - | - | - | - | - | 1 | 0.01% |  |
| CTAG/CTAG | 4 | - | - | - | - | - | - | - | - | - | - | 4 | 0.06% |  |
| CTCA/TGAG | 4 | - | - | - | - | - | - | - | - | - | - | 4 | 0.06% |  |
| CTCC/GGAG | 2 | 1 | - | 1 | - | - | - | - | - | - | - | 4 | 0.06% |  |
| CTCG/CGAG | 1 | 2 | - | - | - | - | - | - | - | - | - | 3 | 0.04% |  |
| CTGA/TCAG | 1 | - | - | - | - | - | - | - | - | - | - | 1 | 0.01% |  |
| CTGG/CCAG | 1 | - | - | - | - | - | - | - | - | - | - | 1 | 0.01% |  |
| GAAA/TTTC | 12 | 2 | - | - | - | - | - | - | - | - | - | 14 | 0.21% |  |
| GAAC/GTTC | 4 | - | - | - | - | - | - | - | - | - | - | 4 | 0.06% |  |
| GAAG/CTTC | 3 | 3 | - | - | - | - | - | - | - | - | - | 6 | 0.09% |  |
| GACA/TGTC | 1 | 1 | - | - | - | - | - | - | - | - | - | 2 | 0.03% |  |
| GATA/TATC | 5 | 2 | - | - | - | - | - | - | - | - | - | 7 | 0.10% |  |
| GATG/CATC | 1 | - | - | - | - | - | - | - | - | - | - | 1 | 0.01% |  |
| GCAA/TTGC | - | 1 | - | - | - | - | - | - | - | - | - | 1 | 0.01% |  |
| GCTG/CAGC | 1 | - | - | - | - | - | - | - | - | - | - | 1 | 0.01% |  |
| GGAA/TTCC | 5 | 1 | - | - | - | - | - | - | - | - | - | 6 | 0.09% |  |
| GTAC/GTAC | 1 | - | - | - | - | - | - | - | - | - | - | 1 | 0.01% |  |
| GTCA/TGAC | 2 | - | - | - | - | - | - | - | - | - | - | 2 | 0.03% |  |
| GTGA/TCAC | 4 | 2 | - | - | - | - | - | - | - | - | - | 6 | 0.09% |  |
| GTGC/GCAC | 6 | - | - | - | - | - | - | - | - | - | - | 6 | 0.09% |  |
| GTTA/TAAC | 1 | - | - | - | - | - | - | - | - | - | - | 1 | 0.01% |  |
| GTTG/CAAC | - | 1 | - | - | - | - | - | - | - | - | - | 1 | 0.01% |  |
| TAAA/TTTA | 6 | - | - | - | - | - | - | - | - | - | - | 4 | 0.10% |  |
| TACA/TGTA | 8 | 1 | - | - | - | - | - | - | - | - | - | 9 | 0.13% |  |
| TAGA/TCTA | 2 | - | - | - | - | - | - | - | - | - | - | 2 | 0.03% |  |
| TAGC/GCTA | 1 | - | - | - | - | - | - | - | - | - | - | 1 | 0.01% |  |
| TATG/CATA | 1 | - | - | - | - | - | - | - | - | - | - | 1 | 0.01% |  |
| TCAT/ATGA | 2 | 1 | - | - | - | - | - | - | - | - | - | 3 | 0.04% |  |
| TCCA/TGGA | 2 | - | - | - | - | - | - | - | - | - | - | 2 | 0.03% |  |
| TGAA/TTCA | 4 | - | - | - | - | - | - | - | - | - | - | 4 | 0.06% |  |
| TGCC/GGCA | 3 | - | - | - | - | - | - | - | - | - | - | 3 | 0.04% |  |
| TGGC/GCCA | 1 | - | - | - | - | - | - | - | - | - | - | 1 | 0.01% |  |
| TTAA/TTAA | 2 | - | - | - | - | - | - | - | - | - | - | 2 | 0.03% |  |
| TTCT/AGAA | 3 | 1 | - | - | - | - | - | - | - | - | - | 4 | 0.06% |  |
| AAACA/TGTTT | - | 1 | - | - | - | - | - | - | - | - | - | 1 | 0.01% |  |
| AGCAT/ATGCT | - | 1 | - | - | - | - | - | - | - | - | - | 1 | 0.01% |  |
| ATCCT/AGGAT | 1 | - | - | - | - | - | - | - | - | - | - | 1 | 0.01% |  |
| ATTAA/TTAAT | 1 | - | - | - | - | - | - | - | - | - | - | 1 | 0.01% |  |
| CTGGC/GCCAG | 1 | - | - | - | - | - | - | - | - | - | - | 1 | 0.01% |  |
| CTTCA/TGAAG | 1 | - | - | - | - | - | - | - | - | - | - | 1 | 0.01% |  |
| GAAAG/CTTTC | - | - | 1 | - | - | - | - | - | - | - | - | 1 | 0.01% |  |
| GAGCC/GGCTC | 1 | - | - | - | - | - | - | - | - | - | - | 1 | 0.01% |  |
| GTTTC/GAAAC | 1 | - | - | - | - | - | - | - | - | - | - | 1 | 0.01% |  |
| TAATT/AATTA | 1 | - | - | - | - | - | - | - | - | - | - | 1 | 0.01% |  |
| TACAT/ATGTA | - | 1 | - | - | - | - | - | - | - | - | - | 1 | 0.01% |  |
| TCTGT/ACAGA | 1 | - | - | - | - | - | - | - | - | - | - | 1 | 0.01% |  |
| TGTTC/GAACA | 1 | - | - | - | - | - | - | - | - | - | - | 1 | 0.01% |  |
| CCATT/AATGG | - | 1 | - | - | - | - | - | - | - | - | - | 1 | 0.01% |  |
| TACTC/GAGTA | 1 | - | - | - | - | - | - | - | - | - | - | 1 | 0.01% |  |
| ATACTC/GAGTAT | - | 1 | - | - | - | - | - | - | - | - | - | 1 | 0.01% |  |
| CAGGCT/AGCCTG | - | - | - | - | 1 | - | - | - | - | - | - | 1 | 0.01% |  |
| CTCCGG/CCGGAG | - | - | - | 1 | - | - | - | - | - | - | - | 1 | 0.01% |  |
| GCCTCA/TGAGGC | 1 | 1 | - | - | - | - | - | - | - | - | - | 2 | 0.03% |  |
| GGTGCC/GGCACC | 1 | - | - | - | - | - | - | - | - | - | - | 1 | 0.01% |  |
| GGTTAA/TTAACC | - | - | - | - | 1 | - | - | - | - | - | - | 1 | 0.01% |  |
| GGTTTG/CAAACC | 1 | - | - | - | - | - | - | - | - | - | - | 1 | 0.01% |  |
| TCGTGC/GCACGA | 1 | - | - | - | - | - | 1 | - | - | - | - | 2 | 0.03% |  |
| TGAACA/TGTTCA |  | 1 | - | - | 1 | - | - | - | - | - | - | 2 | 0.03% |  |
| TTCTCC/GGAGAA | 1 | - | - | - | - | - | - | - | - | - | - | 1 | 0.01% |  |
| TTTTGG/CCAAAA | - | 1 | - | - | - | - | - | - | - | - | - | 1 | 0.01% |  |
| GAACGG/CCGTTC | - | 1 | - | - | - | - | - | - | - | - | - | 1 | 0.01% |  |
| ATAGCC/GGCTAT | 1 | - | - | - | - | - | - | - | - | - | - | 1 | 0.01% |  |
