# Supplementary material for: Development of polymorphic EST-SSR markers and characterization of the autotetraploid genome of sainfoin (Onobrychis viciifolia)
Source: PeerJ. 2019 Mar 26;7:e6542. doi: 10.7717/peerj.6542 (PMC6440460; doi:10.7717/peerj.6542)
Supplement: Table S2 [file peerj-07-6542-s008.docx]

**Supplemental Table S2** **Analysis of 200 EST-SSRs.**

| **Items** | **Number** |
| --- | --- |
| Not amplify | 22 |
| Successfully amplified | 178 |
| Larger or smaller than expected size | 46 |
| Expected size | 132 |
| Polymorphic | 61 |
| Monomorphic | 71 |
| Total | 200 |
