# Supplementary material for: Development of polymorphic EST-SSR markers and characterization of the autotetraploid genome of sainfoin (Onobrychis viciifolia)
Source: PeerJ. 2019 Mar 26;7:e6542. doi: 10.7717/peerj.6542 (PMC6440460; doi:10.7717/peerj.6542)
Supplement: Table S3 [file peerj-07-6542-s009.docx]

**Supplemental Table S3 *Onobrychis viciifolia* accessions used for marker validation.**

| **No.** | **Source** | **Latin name** | **Longitude (E)** | **Latitude (N)** | **Altitude (m)** |
| --- | --- | --- | --- | --- | --- |
| I | Minqin, Gansu | *Onobrychisn viciifolia* | 103°39' | 39°20′ | 1451 |
| II | Huining, Gansu | *Onobrychisn viciifolia* | 104°31′ | 35°33′ | 2025 |
| III | Yuzhong, Gansu | *Onobrychisn viciifolia* | 104°21' | 36°09' | 2881 |
| IV | Jingyuan, Gansu | *Onobrychisn viciifolia* | 104°38' | 36°33' | 2032 |
| V | Maqu, Gansu | *Onobrychisn viciifolia* | 101°39' | 33°30' | 3523 |
