# Supplementary material for: Development of polymorphic EST-SSR markers and characterization of the autotetraploid genome of sainfoin (Onobrychis viciifolia)
Source: PeerJ. 2019 Mar 26;7:e6542. doi: 10.7717/peerj.6542 (PMC6440460; doi:10.7717/peerj.6542)
Supplement: Table S4 [file peerj-07-6542-s010.docx]

**Supplemental Table S4** **Characteristics of the 61 novel EST-SSR markers in *Onobrychis viciifolia*.**

| **Primer** | **Gene_ID** | **Forward primer (5′–3′)** | **Reverse primer (5′–3′)** | **Tm (°C)** | **SSRs** | **Number of alleles** | **Ho** | **He** | **PIC** |
| --- | --- | --- | --- | --- | --- | --- | --- | --- | --- |
| Vo4 | c15761.graph_c0 | CCTTCGTTCATCTTGCTTCC | AGCTCTCTGGGAGGGCTAAG | 56 | TC(2*6) | 8 | 0.85 | 0.79 | 0.76 |
| Vo8 | c16457.graph_c0 | TGTGAAGAAGCTGGCATTTG | TAAAGGCTTCCATTTGGCAT | 51 | CAG(3*5) | 7 | 0.65 | 0.78 | 0.75 |
| Vo9 | c16457.graph_c0 | CACCGTGACATCAAACCAAG | ATAACCAATTGTACCCGGCA | 53 | TCA(3*5) | 6 | 0.25 | 0.65 | 0.61 |
| Vo10 | c17259.graph_c0 | GAAACAAGAACAGAAGCCGC | TTCCACATTTAGGAAACACCA | 52 | CTG(3*6) | 5 | 0.7 | 0.73 | 0.68 |
| Vo12 | c18974.graph_c0 | CTGTCTTGGGGTGGAGCTAT | GTGTTCGCATTGTGGAAAGA | 54 | ATG(3*5) | 6 | 0.72 | 0.74 | 0.7 |
| Vo13 | c18974.graph_c0 | TCTTTCCACAATGCGAACAC | TCTCTCTTTCTCACTCTTACACATACA | 53 | AC(2*6) | 4 | 0.51 | 0.73 | 0.68 |
| Vo15 | c21919.graph_c0 | TCCCTTCAAGGATCCAAAGA | CACTTGCTTTTCTTGCACCA | 52 | ATG(3*5) | 5 | 0.41 | 0.55 | 0.51 |
| Vo18 | c24221.graph_c0 | GAACCTCTTTCACAGCAGCC | ACCAGAGCGCTGAGATCAAT | 55 | CAA(3*6) | 12 | 0.43 | 0.85 | 0.84 |
| Vo20 | c24861.graph_c0 | CAATGGAGGAGAGGTGGAGA | CAACCAAGGCTGGCTAGAAG | 56 | CAA(3*5) | 9 | 0.28 | 0.86 | 0.84 |
| Vo25 | c26396.graph_c0 | TGCTGCAGTTCTTCTGATGG | TGAATTCATGATCATACCCACC | 53 | TAG(3*5) | 6 | 0.46 | 0.64 | 0.61 |
| Vo27 | c27240.graph_c0 | ACCAACACCTCCAAGACCAT | TGGAAGTTTTTCATTCCCGA | 52 | AGA(3*5) | 12 | 0.23 | 0.88 | 0.87 |
| Vo28 | c28110.graph_c0 | AGCAACCCAGAACAACGACT | CATGCGCAAAACTCACTTGT | 53 | GA(2*7) | 9 | 0.78 | 0.84 | 0.82 |
| Vo29 | c28476.graph_c0 | TTTCCAAATCCACCAACTCC | CCAGGGTCAGTCACACACAC | 55 | TAC(3*5) | 9 | 0.95 | 0.87 | 0.86 |
| Vo35 | c28713.graph_c0 | ATCGACACCGACATAGTCCC | TTATGCAAGCTTTGAATGCG | 53 | ATC(3*5) | 5 | 0.68 | 0.62 | 0.57 |
| Vo40 | c29611.graph_c0 | TGCAGACAATAAAAGCGTCG | GTTCTGTCATGGAGGGTGCT | 54 | TC(2*9) | 11 | 0.8 | 0.85 | 0.83 |
| Vo42 | c29611.graph_c0 | AGATTCAACCCTCTCCGGTT | AAAAGCCTTCGTGGTCATTG | 53 | TAG(3*5) | 5 | 0.35 | 0.69 | 0.64 |
| Vo43 | c29626.graph_c0 | TTGCCCGCAAAACTTATAGC | ACACTTTCTTGCCAAAGGGA | 52 | GGT(3*6) | 8 | 1 | 0.81 | 0.79 |
| Vo50 | c30351.graph_c0 | CGTGGTGGTGGTCTTTTTCT | TTATTTGTATGGGGAAGGCG | 53 | GGT(3*5) | 7 | 0.15 | 0.76 | 0.73 |
| Vo55 | c30818.graph_c0 | GACTGTTACGGGCAACCACT | CCCAATACAAACAAGCAGCA | 54 | GGA(3*5) | 5 | 0.2 | 0.71 | 0.66 |
| Vo57 | c31059.graph_c0 | GTTGCTGCAATTGTCCTTGA | TATTTTATTTCCCCACCCCC | 52 | GTG(3*5) | 7 | 0.85 | 0.73 | 0.69 |
| Vo59 | c31137.graph_c0 | CGCTTGTTCTTGAATTTCTGC | TGTGTTGGAAATGGATTGGA | 51 | TTA(3*5) | 5 | 0.85 | 0.72 | 0.67 |
| Vo61 | c31246.graph_c0 | CCGTGTTCCGTAACCTCATC | CCTCGGACTCTCTCCTCTCA | 57 | AGAA(4*6) | 15 | 0.97 | 0.91 | 0.9 |
| Vo65 | c31324.graph_c0 | GGCCTCCTTCTCACTCCTCT | TCTGCAGAATCACAAACAAAGG | 55 | AGT(3*5) | 12 | 0.8 | 0.82 | 0.8 |
| Vo68 | c31609.graph_c0 | TTGGCTTTGTTAATAGATACCTGTC | TCGTTGCTTCTGAGGGATTC | 53 | TTG(3*5) | 8 | 0.98 | 0.83 | 0.81 |
| Vo73 | c31724.graph_c0 | GCGAATGTGTCCTTCACCTT | GTGCCCAGAAGAAAAGCAAG | 54 | TTC(3*5) | 9 | 0.7 | 0.84 | 0.82 |
| Vo76 | c31823.graph_c0 | CTCTCAACAGCAGGAGACCC | ACTAGTGAATGGGGCTGCAC | 57 | TGAACA(6*6) | 6 | 0.75 | 0.72 | 0.68 |
| Vo81 | c31956.graph_c0 | ACCGGTGCCTCTTAATGTGA | TTGCCATCGGATTTTACACA | 52 | GAT(3*5) | 7 | 0.78 | 0.84 | 0.82 |
| Vo82 | c31963.graph_c0 | GAAGAAACTTCCGATCGCTG | CAATGTCGTCTTCATCAGAAGG | 54 | TGA(3*5) | 7 | 0.68 | 0.78 | 0.74 |
| Vo89 | c32454.graph_c0 | GCAAACGAAAATTTGGGAAA | GTGCGGAGGTTTTTGTTGTC | 51 | TCC(3*5) | 7 | 0.68 | 0.81 | 0.78 |
| Vo90 | c32466.graph_c0 | ATTGAGGCTTGCCAAAGAAA | CCACACTCCTTACCAAAGGG | 53 | TAT(3*5) | 8 | 0.68 | 0.8 | 0.78 |
| Vo92 | c32538.graph_c0 | CAGGTCGGAAGATTCCATGT | TTTCGGTTTTGCGCTATGTT | 52 | GA(2*6) | 6 | 0.53 | 0.79 | 0.76 |
| Vo93 | c32552.graph_c0 | CCTTTTTGTTGCTCTCCAAGG | TCGATCTTCGACAAATTCCA | 52 | TGT(3*5) | 11 | 0.73 | 0.86 | 0.85 |
| Vo97 | c32714.graph_c0 | GCACCTAGCTCAGCCATCTC | GGAGGCCGAATACTTCATCA | 56 | CAT(3*5) | 5 | 0.9 | 0.67 | 0.61 |
| Vo100 | c32787.graph_c0 | AGGGGATTTCATGGAGGAAT | TGTGGTGTGGTGTAGGATGG | 54 | TCT(3*5) | 10 | 0.43 | 0.86 | 0.84 |
| Vo102 | c32807.graph_c0 | GTTTCGTCGAAGCCAAATTC | ACTGGGAAGCAGTAGCAGGA | 54 | TTC(3*5) | 10 | 0.88 | 0.87 | 0.86 |
| Vo103 | c32852.graph_c0 | TCCTTTTAGCTCAATGAAGATCG | CAACTGGAGAGGGAGCTGTC | 55 | TA(2*8) | 4 | 0.75 | 0.69 | 0.63 |
| Vo106 | c33087.graph_c0 | AAGCTTGGATTGTTGCATCC | TGTTGCACAGAAGGCTGAAG | 53 | GTA(3*5) | 4 | 0.29 | 0.71 | 0.66 |
| Vo108 | c33178.graph_c0 | TTAATCAAGAAGGGGCAACG | CATTACTTGCTGCAATCACCA | 52 | GAG(3*6) | 6 | 0.24 | 0.8 | 0.77 |
| Vo109 | c33178.graph_c0 | TTTGGATGGTTGAGTTTCAGC | GCTAGCTAGTTTCTTTCCTACTCCA | 54 | GA(2*7) | 11 | 0.45 | 0.86 | 0.84 |
| Vo111 | c33210.graph_c0 | CTTCCTCCTGGCTGACACTC | TACGATCGCGATGAATCAAG | 55 | CTA(3*5) | 9 | 0.79 | 0.8 | 0.81 |
| Vo115 | c33340.graph_c0 | TGATGCGTGTCCTAACCAAA | TGATCCAAACCCATCGAACT | 52 | AGT(3*6) | 9 | 0.72 | 0.82 | 0.8 |
| Vo117 | c33423.graph_c0 | GGAGCCGATTCCAAGAAAC | CTCAGATACGACCCCTGGAA | 55 | GTTA(4*5) | 5 | 0.05 | 0.6 | 0.54 |
| Vo118 | c33433.graph_c0 | TTTTCTCCTTCGTCCCATTG | GGAGTTGTTCGACGGGTAAA | 53 | AGC(3*5) | 6 | 0.88 | 0.8 | 0.77 |
| Vo119 | c33452.graph_c0 | AGACTACGCGCATCAAAATG | TGGTTTGGTTTGGTTTGGTT | 51 | TTC(3*5) | 11 | 0.56 | 0.87 | 0.86 |
| Vo121 | c33532.graph_c0 | TTCATCTTCAACCTCCTGGC | TTTCAACGTTGTGAAGAGCG | 53 | ATC(3*5) | 11 | 0.98 | 0.86 | 0.85 |
| Vo122 | c33537.graph_c0 | TGACACCCCAAAAATACCCA | TTTCATCATCTTGAAGGAATTGA | 50 | ATT(3*5) | 6 | 0.55 | 0.68 | 0.63 |
| Vo125 | c33552.graph_c0 | TCCTGACACAGAAGGCTTGA | CTTTCTCCCTTCACACACCC | 55 | TG(2*7) | 11 | 0.93 | 0.85 | 0.83 |
| Vo126 | c33560.graph_c0 | AAAGACCAGCAGAGGGGACT | TCAAAACACAACCAGCACCT | 54 | TGA(3*6) | 9 | 0.93 | 0.8 | 0.77 |
| Vo127 | c33567.graph_c0 | AAAAGGGATGCAATTGTTGG | TGGCCTGGAATTGAGGATAA | 51 | GAC(3*5) | 9 | 0.98 | 0.86 | 0.84 |
| Vo129 | c33590.graph_c0 | CTCTTCCCGGAGTTCATCAG | TGATGACGAGAAGACGATGG | 55 | GAT(3*5) | 7 | 0.49 | 0.78 | 0.75 |
| Vo133 | c33611.graph_c0 | TGTGAGAATGCTTCCGTCAG | CCTTCCCAAGTCATACCCAA | 54 | GAG(3*5) | 8 | 0.93 | 0.86 | 0.84 |
| Vo135 | c33623.graph_c0 | CCTTCCTTCTATCCCCAACC | AGCATCTGTGTCTGTGACGG | 56 | TC(2*9) | 7 | 0.41 | 0.74 | 0.71 |
| Vo137 | c33633.graph_c0 | AGGAAGGGAAGATAGCCCTG | TCATCTCCGGTGGAAAACTC | 55 | GT(2*6) | 8 | 0.89 | 0.76 | 0.73 |
| Vo139 | c33648.graph_c0 | AGCCCCCGCGATAGATAATA | CCCTCCTCCTTGTTTCTTCC | 55 | CA(2*7) | 4 | 0.82 | 0.66 | 0.6 |
| Vo145 | c33755.graph_c1 | TGGAGTTGGAGTTGGAGGAG | GCGTCTGTGTTGTTGTCGAT | 55 | GAT(3*6) | 4 | 0.78 | 0.68 | 0.62 |
| Vo157 | c33977.graph_c0 | CAACAACCAAACCCGATTTC | CAAACCTTGAAACCGTTCGT | 52 | ACC(3*5) | 12 | 0.8 | 0.89 | 0.88 |
| Vo159 | c34000.graph_c0 | GGTCGAAAATGCTGAGCTTC | CTTTGCCATCATCACCACTG | 54 | CTT(3*5) | 6 | 0.93 | 0.71 | 0.66 |
| Vo164 | c34108.graph_c0 | ATGGAGGAGGAAGAGGTGGT | TTAATGGTTACCGCGGTTTG | 54 | CTT(3*5) | 4 | 0.72 | 0.72 | 0.67 |
| Vo178 | c34312.graph_c0 | ATGGTGTGGCTTTCTTCCAG | GGGAGAAATCGAGGAAAAGG | 54 | ACG(3*7) | 5 | 0.79 | 0.71 | 0.66 |
| Vo182 | c34436.graph_c0 | AATGTGAAGGGTGAATCCCA | AGCTTGGAGCTTTGAAGTCG | 53 | TC(2*7) | 6 | 0.83 | 0.79 | 0.76 |
| Vo194 | c34731.graph_c0 | GGTTTCGGAGGAACATTCAA | CGAGACATTCACGCCTTCTT | 53 | TGT(3*5) | 5 | 0.73 | 0.72 | 0.68 |
| Mean± sd | - | - | - | - | - | 7.52±2.57 | 0.67±0.2 | 0.77±0.1 | 0.74±0.1 |
