# Supplementary material for: Development of polymorphic EST-SSR markers and characterization of the autotetraploid genome of sainfoin (Onobrychis viciifolia)
Source: PeerJ. 2019 Mar 26;7:e6542. doi: 10.7717/peerj.6542 (PMC6440460; doi:10.7717/peerj.6542)
Supplement: Table S5 [file peerj-07-6542-s011.docx]

**Supplemental Table S5 The types and distributions potential EST-SSRs**

| **Searching Item** | **Number** |
| --- | --- |
| Total number of sequences examined | 14,852 |
| Total size of examined sequences (bp) | 29,374,998 |
| Total number of identified SSRs | 6,752 |
| Number of SSR containing sequences | 4,988 |
| Number of sequences containing more than 1 SSR | 1,271 |
| Number of SSRs present in compound formation | 415 |
| Mono nucleotide | 2,906 |
| Di nucleotide | 1,287 |
| Tri nucleotide | 2,262 |
| Tetra nucleotide | 263 |
| Penta nucleotide | 15 |
| Hexa nucleotide | 19 |
