# Supplementary material for: Development of polymorphic EST-SSR markers and characterization of the autotetraploid genome of sainfoin (Onobrychis viciifolia)
Source: PeerJ. 2019 Mar 26;7:e6542. doi: 10.7717/peerj.6542 (PMC6440460; doi:10.7717/peerj.6542)
Supplement: Table S6 [file peerj-07-6542-s012.docx]

**Supplemental Table S6** **Population analysis data in the structure.**

| **K** | **2** | **3** | **4** | **5** | **6** | **7** | **8** |
| --- | --- | --- | --- | --- | --- | --- | --- |
| Ln P(D) | -15561.1 | -14835.4 | -14038.0 | -13599.0 | -13190.2 | -12822.0 | -18338.5 |
| ΔK | - | 0.078 | 0.312 | 0.096 | 144.000 | 5.632 | - |
